# The role of [^18^F]fluorodopa positron emission tomography in grading of gliomas

**DOI:** 10.1007/s11060-022-04177-3

**Published:** 2022-11-24

**Authors:** Joy R. Roach, Puneet Plaha, Daniel R. McGowan, Geoff S. Higgins

**Affiliations:** 1 Department of Oncology, University of Oxford, Oxford, OX3 7DQ UK; 2 Department of Neurosurgery, Oxford University Hospital NHS FT, John Radcliffe Hospital, L3 West Wing, Oxford, OX3 9DU UK; 3 Nuffield Department of Surgical Sciences, University of Oxford, Oxford, OX3 7DQ UK; 4 Department of Medical Physics and Clinical Engineering, Oxford University Hospital NHS FT, Churchill Hospital, Oxford, OX3 7LE UK; 5 Department of Oncology, Oxford University Hospitals NHS FT, Oxford, UK

**Keywords:** Gliomas, Glioma grading, FDOPA PET, Fluorodopa pet, Brain tumours

## Abstract

**Purpose:**

Gliomas are the most commonly occurring brain tumour in adults and there remains no cure for these tumours with treatment strategies being based on tumour grade. All treatment options aim to prolong survival, maintain quality of life and slow the inevitable progression from low-grade to high-grade. Despite imaging advancements, the only reliable method to grade a glioma is to perform a biopsy, and even this is fraught with errors associated with under grading. Positron emission tomography (PET) imaging with amino acid tracers such as [^18^F]fluorodopa (^18^F-FDOPA), [^11^C]methionine (^11^C-MET), [^18^F]fluoroethyltyrosine (^18^F-FET), and ^18^F-FDOPA are being increasingly used in the diagnosis and management of gliomas.

**Methods:**

In this review we discuss the literature available on the ability of ^18^F-FDOPA-PET to distinguish low- from high-grade in newly diagnosed gliomas.

**Results:**

In 2016 the Response Assessment in Neuro-Oncology (RANO) and European Association for Neuro-Oncology (EANO) published recommendations on the clinical use of PET imaging in gliomas. However, since these recommendations there have been a number of studies performed looking at whether ^18^F-FDOPA-PET can identify areas of high-grade transformation before the typical radiological features of transformation such as contrast enhancement are visible on standard magnetic resonance imaging (MRI).

**Conclusion:**

Larger studies are needed to validate ^18^F-FDOPA-PET as a non-invasive marker of glioma grade and prediction of tumour molecular characteristics which could guide decisions surrounding surgical resection.

## Introduction

Glioma is the most common primary brain tumour occurring in adults for which there is no cure. Gliomas are traditionally dichotomised by their grade into low-grade gliomas (LGG) which include grade I and II and high-grade gliomas (HGG) which includes grade III and IV tumours. The most recent publication of the fifth edition of the WHO classification of brain tumours incorporates new information on histological and molecular features into a layered integrated diagnosis and also introduces grading on a ‘within tumour type’ method [1].

The majority of patients with a low-grade glioma will present acutely with a seizure and as a result have a plain or contrast-enhanced computed tomography (CT) brain scan which identifies an area of abnormality that then requires further delineation with magnetic resonance imaging (MRI). The minimum MRI sequences performed as part of the diagnostic workup for a suspected brain tumour include T2 weighted, fluid attenuated inversion recovery (FLAIR), T1 pre- and post-contrast and diffusion weighted sequences (DWI). The characteristic appearances of low-grade gliomas on MRI sequences will grossly depend on the grade and histological type of glioma. A provisional diagnosis can be made on imaging alone i.e. distinguishing between glioma versus metastasis, and within gliomas in distinguishing low-grade versus high-grade depending on a number of tumour characteristics [2]. However, there is diagnostic uncertainty about the prediction of WHO grade, and imaging alone is often inaccurate.

The distinction between low- and high-grade glioma on MRI is based on contrast enhancement from a disrupted blood brain barrier and to some extent from diffusion characteristics but this is not always truly predictive of grade and there is often diagnostic uncertainty. The confirmation of grade is normally made on biopsy sampling. However, sampling errors are not uncommon and up to one third of high-grade gliomas may not display the typical imaging characteristics of a high-grade glioma with enhancement [3]. Under grading is particularly associated with large heterogenous tumours and has been reported in 28% – 68% of cases [4–6]. With the introduction of molecular characterisation the risk of sample bias has reduced dramatically as the molecular markers are volume independent but not completely eliminated [7].

Additional imaging techniques and sequences are frequently used to provide additional information to help distinguish the type, grade of tumour and predict transformation. These include positron emission tomography (PET), single photon emission computed tomography (SPECT), MR perfusion and MR spectroscopy (MRS). Gliomas are notoriously heterogeneous in nature and as a result the use of MRS will demonstrate spectra that vary significantly depending on the region sampled [8]. The use of MR perfusion to detect grade has been demonstrated with varying results [9–11]. SPECT imaging has the disadvantage over PET of a lower resolution [12]. PET using amino acid tracers is becoming increasingly common to differentiate gliomas from metastases or other types of tumours [13]. The most commonly used amino acid PET tracers described are [^11^C]methionine (^11^C-MET), [^18^F]fluoroethyltyrosine (^18^F-FET) and [^18^F]fluorodopa (^18^F-FDOPA). ^11^C-MET has a short half-life of 20 min which limits its use to centres that have an onsite cyclotron. In comparison ^18^F-FET and ^18^F-FDOPA have much longer half-lives of 110 min making these tracers more available for clinical use. ^18^F-FDOPA demonstrates greater contrast for lesions outside of the striatum when compared to^18^F-FET [14]. A type of novel MRI technique, called oxygen enhanced MRI (OEMRI), has the ability to detect areas of hypoxia within solid tumours which if used within glioma could potentially detect areas of high-grade tumour and therefore affect prognosis if detected earlier [15].

**Fig. 1:**
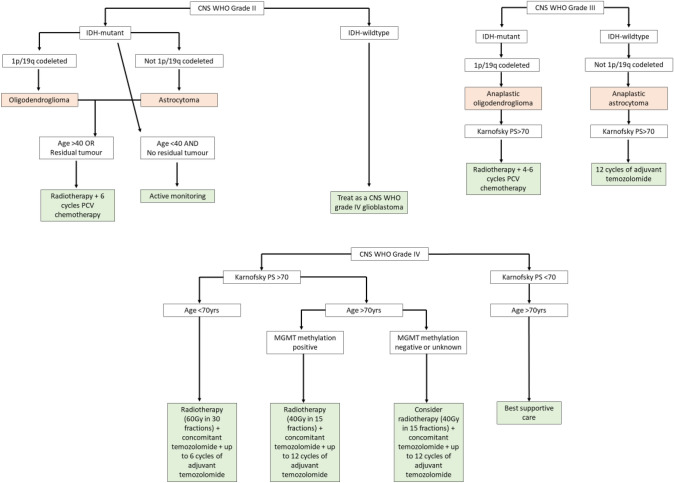
NICE guidelines for management of glioma following biopsy or surgical resection

The distinction between high-grade and low-grade glioma is important as both entities confer very different prognoses and management strategies. All treatment options aim to prolong survival, maintain quality of life and slow the inevitable progression from low- to high-grade. For both low- and high-grade gliomas the NICE guidelines recommend consideration of maximum safe gross total resection to confirm the histological and molecular diagnosis and for tumours that are not surgically resectable to perform a biopsy [16]. Following surgery, the standard treatment regime for grade IV gliomas (glioblastoma) is radiotherapy with concomitant temozolomide followed by adjuvant temozolomide. In patients over the age of 70 treatment for grade IV gliomas is hypofractionated chemoradiotherapy [16]. Even with full treatment the median survival for grade IV glioblastomas is 12–24 months [17]. For grade II gliomas following surgery, oncological therapy is dependent on the patient’s age, extent of resection, 1p/19q codeletion presence and isocitrate dehydrogenase (IDH) mutation status and consists of a combination of radiotherapy and PCV (procarbazine, CCNU [lomustine] and vincristine) chemotherapy [16] (Fig. 1). For grade III gliomas with a 1p/19q codeletion (anaplastic oligodendroglioma) treatment is similar with radiotherapy and PCV chemotherapy. Grade III tumours without a 1p/19q codeletion (anaplastic astrocytoma) require radiotherapy followed by adjuvant temozolomide. The use of non-invasive imaging parameters to accurately detect glioma grade could aid preoperative clinical decision-making when considering biopsy versus resection, extent of resection and timing of surgery.

## Current recommendations

In 2016, the European Association of Nuclear Medicine (EANM), European Association of Neurooncology (EANO) and the Response Assessment in Neurooncology (RANO) working group jointly published guidelines on the role of amino acid PET for imaging in gliomas [18, 19]. These evidence-based guidelines recommended a clinical role for the tracer ^18^F-FDOPA in the differentiation of glioma recurrence from treatment-induced changes, assessment of treatment response and assessment of prognosis. The recommendations reviewed the literature on the amino acid tracers ^11^C-MET, ^18^F-FET and ^18^F-FDOPA in glioma. Multiple studies have found that response to treatment is indicated on PET imaging by a decrease in amino acid tracer uptake with or without a reduction in the volume of metabolically active tumour [20–22] and ^18^F-FDOPA has been shown to demonstrate response to bevacizumab therapy better than conventional MRI [23, 24]. Treatment of gliomas with radiotherapy and/or chemotherapy can result in treatment-related changes. There is a temporary alteration in the blood brain barrier (BBB) resulting in contrast enhancement on MRI imaging which mimics tumour progression and is called pseudoprogression. Pseudoprogression typically occurs within 12 weeks of completion of treatment [25, 26]. Differentiating pseudoprogression and radionecrosis from true tumour progression can be challenging with conventional MRI alone and often additional imaging techniques such as amino acid PET or MR spectroscopy are employed [27]. In a prospective study of 35 patients with proven glioma, Karunanithi et al. found the sensitivity of ^18^F-FDOPA-PET/CT in determining recurrence in glioma to be 100% when compared to 92% with contrast-enhanced MRI and a specificity of 89% with ^18^F-FDOPA-PET/CT versus 44% for contrast enhanced-MRI [28]. Karunanithi et al. in a separate study of 28 patients with proven glioma compared ^18^F-FDOPA-PET/CT with ^18^F-Fluoro-deoxy-glucose (FDG)-PET/CT and found the sensitivity and specificity for FDG was inferior at 48% and 100% respectively and in comparison, ^18^F-FDOPA-PET/CT was 100% and 86% respectively [29]. A meta-analysis by de Zwart et al. found ^18^F-FDOPA to be superior over ^11^C-MET and ^18^F-FET in differentiating tumour progression from treatment-related changes with a pooled sensitivity of 85–100% and specificity of 72–100% when compared to ^11^C-MET (sensitivity 80–98%, specificity 61–91%) and ^18^F-FET (sensitivity 81–95%, specificity 71–93%) [30]. A study by Villani et al. with 50 patients found a potential role of FDOPA in prognostication of low-grade gliomas [31]. The authors found that disease duration and a maximum standardised uptake value (SUV_max_) of > 1.75 was predictive of progression and superior to MRI in detection of progression and therefore prognosis, as transformation to high-grade is the key determinant in patient survival. When the recommendations were published there were conflicting results on the ability of ^18^F-FDOPA-PET to predict glioma grade and ^18^F-FDOPA was not recommended for this use. This article will review previously published results and results from recent studies that have become available since the publication of these guidelines.

## ^18^F-FDOPA PET

**Fig. 2:**
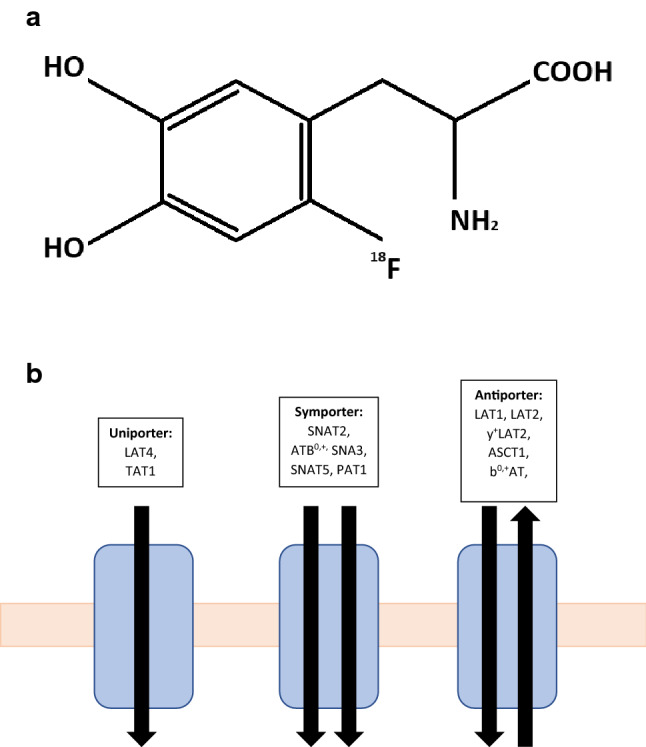
**a** Chemical structure of [^18^F]fluorodopa (–NH_2_ = amine group, –COOH = carboxyl group). **b** Amino acids reach tumour cells via amino acid transporters. Amino acid transporters can be uniporter, symporter or antiporter and each have carrier numbers as demonstrated

**Fig. 3:**
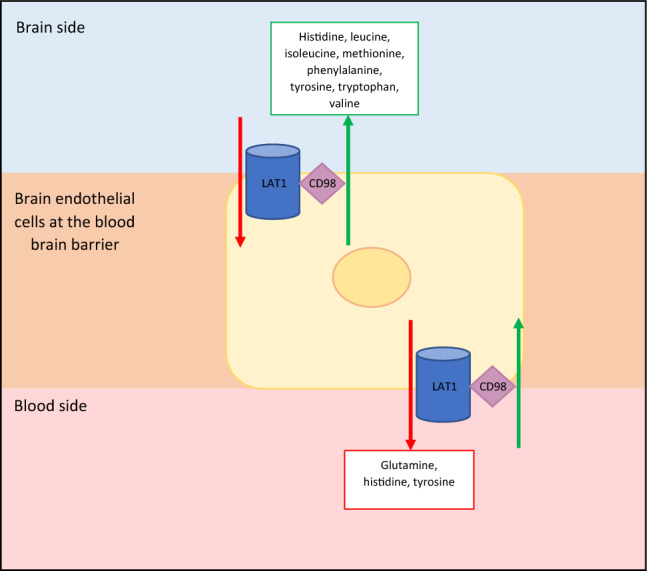
Structure and function of the LAT1 amino acid transporter

[^18^F]fluorodopa, 3, 4-dihydroxy-6-[^18^F]fluoro-L-phenylalanine, (^18^F-FDOPA) was originally developed for brain imaging in patients with movement disorders. It consists of an amino acid, phenylalanine, attached to a radioisotope, fluorine (Fig. 2a), that is able to cross the blood brain barrier and act as a precursor for dopamine. Phenylalanine is an essential aromatic amino acid with a neutral charge. Gliomas require a continuous supply of amino acids to maintain protein synthesis and cell proliferation. These amino acids reach the tumour cells via amino acid transporters. Amino acids are cationic, anionic or neutral, and their transport across a membrane is regulated by amino acid transporters [32]. Amino acid transporters can be uniporter, antiporter or symporter, each transporting certain amino acids (Fig. 2b). One of these neutral transporter systems is the L-type amino acid transporter (LAT) which is a membrane bound Na^+^ independent transport protein regulating the transport of essential amino acids across cell membranes. There are four main types of LAT transporter; LAT1 (SLC7A5), LAT2 (SLC7A8), LAT3 (SLC43A1) and LAT4 (SLC43A2). LAT1 is a polypeptide consisting of 507 amino acids and 12 transmembrane regions with a molecular weight of 55 kDa [33]. LAT1 forms a heterodimeric complex with CD98 via a disulphide bond. CD98 is a polypeptide of 630 amino acids and a molecular weight of 68 kDa. CD98 is thought to be crucial for LAT1 to function but it has been demonstrated that LAT1 is the only transport component of the LAT1/CD98 heterodimer [33, 34]. Substrates that bind the LAT1 transporter must have a histidine group, a carboxylic group and an amino group [35]. LAT1 controls the movement of neutral essential amino acids which include histidine, leucine, isoleucine, methionine, phenylalanine, tyrosine, tryptophan and valine, into cells in exchange for the efflux of intracellular substrates such as glutamine, histidine and tyrosine (Fig. 3) [36, 37]. LAT1 mRNA is expressed most strongly in the brain, placenta, colon, testis and spleen and is expressed at low levels in the lungs, liver and heart [38]. Studies have demonstrated that LAT1 expression is higher than in normal tissues in cancers [39]. Tumour progression in gliomas requires a constant supply of amino acids for protein synthesis and cell proliferation. LAT1 expression correlates with proliferation of cancer cells enabling rapid growth as it plays role in cell growth, transcription and translation through the mammalian target of rapamycin (mTOR) signalling pathway which facilitates protein synthesis [35, 40].

**Fig. 4:**
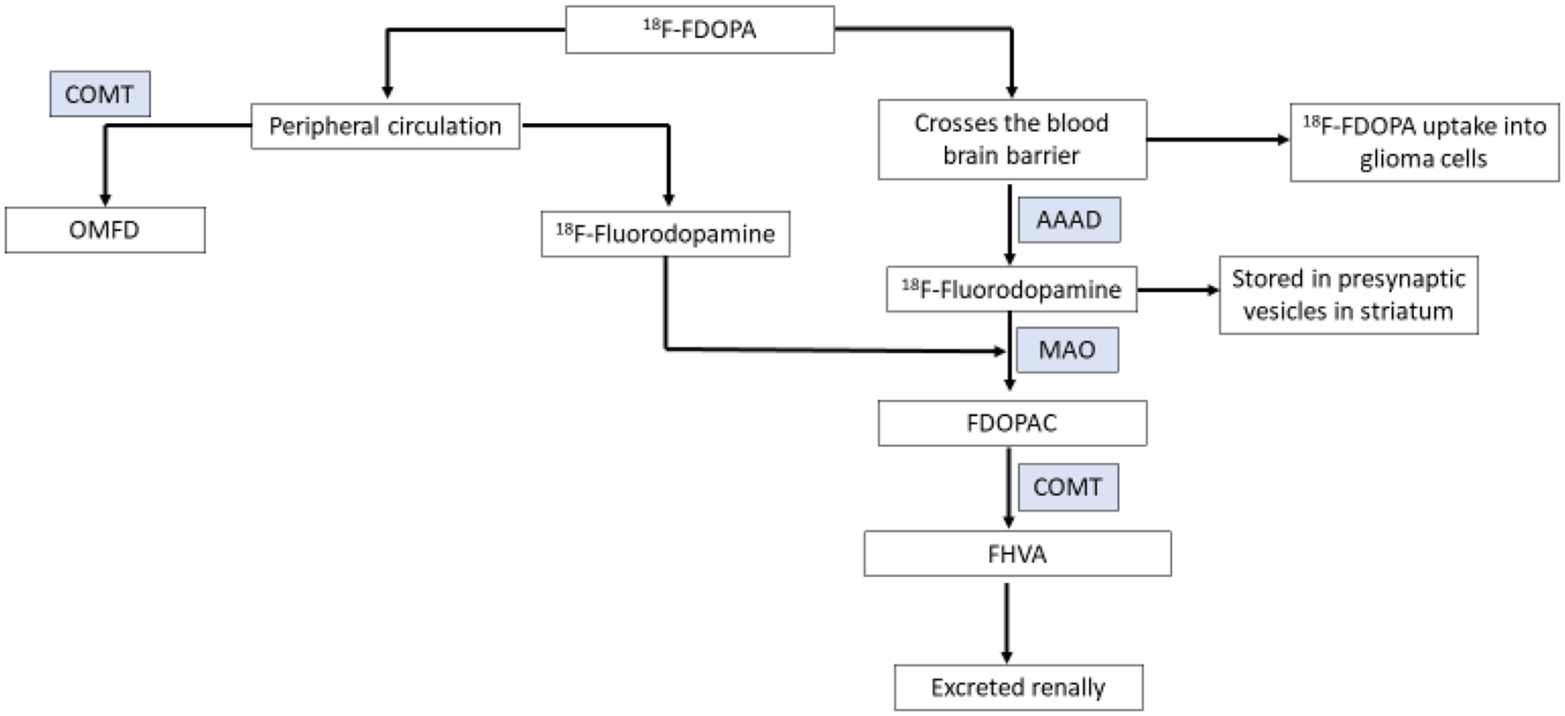
Metabolism of FDOPA (AAAD = aromatic amino acid decarboxylase, FDA = 18F6-dlurodopamine, COMT = catechol O-methyl transferase, OMFD = 3-O-methyl-6-fluoro-L-DOPA, FDOPAC = [^18^F]6-fluoro-L-3,4-dihydrophenylacetic acid, MAO = monoamine oxidase (MAO), FHVA = [^18^F]6-fluorohomovanillic acid)

When administered intravenously, 1% of the ^18^F-FDOPA will cross the blood brain barrier via the LAT1 transporter and the rest remains in the periphery where it is converted to 3-O-methyl-6-fluoro-L-DOPA (OMFD) by catechol O-methyl transferase (COMT) or into [^18^F]fluorodopamine by aromatic amino acid decarboxylase (AAAD). Once across the BBB FDOPA is converted to [^18^F]fluorodopamine by aromatic amino acid decarboxylase (AAAD) (Fig. 4). Fluorodopamine behaves like dopamine in vivo and is either stored in pre-synaptic vesicles in the striatum or metabolised into [^18^F]6-fluoro-L-3,4-dihydrophenylacetic acid (FDOPAC) by monoamine oxidase (MAO) and then into [^18^F]6-fluorohomovanillic acid (FHVA) by COMT [41]. The ^18^F-FDOPA metabolites are renally excreted. In glioma cells the ^18^F-FDOPA is not metabolised [42]. To reduce the systemic metabolism of ^18^F-FDOPA and increase bioavailability and cerebral uptake, the decarboxylase inhibitor carbidopa is often administered prior to administration of ^18^F-FDOPA. Bros et al. found that in imaging of gliomas with ^18^F-FDOPA-PET, premedication with carbidopa resulted in a 50% increase in uptake in all brain structures but when corrected for the tumour-to-healthy-brain ratio did not impact image interpretation [43].

^18^F-FDOPA has been demonstrated to be advantageous over other amino acid PET tracers as it is predominantly transported by the L-type amino acid transporter without significant uptake into surrounding normal brain parenchyma with the exception of the basal ganglia, thereby allowing easier discrimination of uptake within the tumour [44, 45]. Despite this limitation, in gliomas involving the basal ganglia ^18^F-FDOPA has been shown be able to accurately delineate tumour boundaries [46, 47]. ^18^F-FDOPA is more readily available in clinical practice compared to ^11^C-MET which requires an on-site cyclotron due to its short half-life of 20 minutes whereas the half-life of ^18^F-FDOPA is 110 minutes [48]. ^18^FDG has been extensively used for imaging in brain tumours with a meta-analysis of 26 studies that reported a sensitivity of 77% and specificity of 78% in detecting gliomas irrespective of grade [49–51]. However, ^18^FDG measures glucose metabolism and therefore it tends to accumulate in the grey matter which can interfere with the ability to differentiate tumour grade reliably. Tumours with low glucose metabolism, such as low-grade gliomas, are often not well visualised with FDG [52]. ^18^F-FDOPA has been shown to have a higher uptake in low-grade gliomas than FDG [53]. ^18^F-FDOPA uptake into glioma cells is thought to be higher in areas with high-grade features due to an increase in the transport of amino acids into tumour cells which is led by an increase in expression of the L-type amino acid transport system and subsequently has the potential to be able to detect areas of high-grade transformation within low-grade glioma before the typical radiological features of transformation such as contrast enhancement are visible on conventional MRI [54, 55]. Ledezma et al. found in a small number of cases that ^18^F-FDOPA tracer activity was able to identify tumour not visible on conventional MRI [52].

## Role of ^18^F-FDOPA in differentiating high- and low-grade gliomas

The standardised uptake value (SUV) is a measure of ^18^F-FDOPA uptake and is a calculation of the ratio of tissue radioactivity concentration (in kBq/ml) at a given time divided by the administered activity at the time of injection (in MBq) divided by the body weight (in kg) [56]. Despite studies investigating ^18^F-FDOPA SUV in differing grades of glioma there are currently no agreed thresholds for SUV in routine clinical practice with ^18^F-FDOPA for distinguishing between high and low-grade gliomas. The Joint EANM/EANO/RANO guidelines do however provide thresholds for ^18^F-FDOPA in delineating tumour extent, detecting tumour recurrence and identification of response to treatment with bevacizumab [18]. For extent of tumour Pafundi et al. identified that a tumour-to-normal-brain ratio (TBR) of greater than 2.0 corresponded to high-grade disease [57]. For detection of tumour recurrence, a tumour to striatum ratio (TSR) max of 2.1 and a TSR_mean_ of 1.8 are described [58]. Scwharzenberg et al. were able to show that by using ^18^F-FDOPA-PET at two weeks following initiation of bevacizumab therapy for recurrent high-grade gliomas the threshold for a positive response to treatment was found to be a brain tumour volume decrease of greater than 35% or a metabolically active tumour volume of less than 18 mL at two weeks [24].

### Literature published prior to joint EANM/EANO/RANO guidelines

Prior to the publication of the 2016 recommendations there were two main studies that have addressed ^18^F-FDOPA PET in predicting glioma grade. The first was by Pafundi et al. who performed a prospective pilot study with 10 patients [57]. Of these, 8 were newly diagnosed and 2 were recurrent gliomas. For the patients undergoing surgical resection, a maximum of three stereotactic biopsy targets were planned using various PET SUVs along a single trajectory with the PET/CT and contrast enhanced MRI fused using MIM Maestro software. 23 biopsy samples were obtained using neuronavigation on the preplanned targets and each tissue sample was analysed and graded along with recording the average cellularity and average Ki-67 index. PET/CT was performed 10 min after the tracer injection and carbidopa premedication was not used. A strong association was found of ^18^F-FDOPA SUV_max_ with tumour grade. The authors identified that there were significant differences between distinguishing grade II and IV (p = 0.008) and grade III and IV (p = 0.024) when using static ^18^F-FDOPA-PET/CT in 8 patients. No significant difference was found between the SUV_max_ in grade II and grade III tumours (p = 0.17). This may have been because there were only two grade II astrocytomas in the cohort. By removing the oligodendroglioma samples the authors found a significant correlation between ^18^F-FDOPA SUV_mean_ and histological cellularity (p = 0.01). Higher cellularity indicates histologically higher-grade features. A TBR of > 2.0 was able to define high-grade components of astrocytic tumours. The oligodendroglioma biopsy samples, of which there were three, were removed from the final analysis as the study found that the ^18^F-FDOPA SUV_max_ was much greater in comparison to the grade II astrocytomas and this has been previously described with the tracers ^11^C-MET^55^ and ^18^F-FET [60].

The second study by Fueger et al. included 59 patients of which 22 where newly diagnosed and 37 recurrent gliomas of varying grades (grade II n = 22, grade III n = 19, grade IV n = 27) that underwent static ^18^F-FDOPA-PET/CT before surgery [61]. The PET/CT was performed 10 min after the tracer injection and carbidopa premedication was not administered. Each tumour was graded histologically and Ki-67 expression measured. The authors found a significant difference in SUV_max_ in newly diagnosed gliomas between grade II and III (p = 0.044), between grade II and IV (p = 0.007) and between grade III and IV tumours (p = 0.010) and concluded that a ^18^F-FDOPA SUV_max_ of 2.72 was the cut-off to distinguish low- and high-grade newly diagnosed gliomas. There was no significant difference between grades in the recurrent tumours. The lack of correlation in tumour recurrence could be explained by damage to the BBB from radiation therapy leading to increased vascular permeability resulting in an increase in non-carrier (LAT1) mediated transport of ^18^F-FDOPA from endothelial cells into tumour cells [62]. The histopathological samples from this study were much larger compared to Pafundi et al. [57] and therefore would have not considered ^18^F-FDOPA uptake heterogeneity within the tumour.

### Literature published after Joint EANM/EANO/RANO guidelines available

The following section will describe the new data published following the 2016 recommendations and the impact of this data. Todeschi et al. recently performed a single centre prospective study on 16 newly diagnosed gliomas and 4 recurrent gliomas using static ^18^F-FDOPA-PET/CT [63]. The PET/CT was performed 30 min after tracer injection and no carbidopa premedication was administered. Biopsy targets were based on regions of tracer hypermetabolism and hypometabolism. In this series the authors found that a SUV_max_ threshold of > 1.75 demonstrated a greater yield in terms of diagnosis of high-grade gliomas. The authors concluded that the use of ^18^F-FDOPA-PET allowed for better targeting of metabolically active areas of tumour to reduce biopsy sampling bias but when used alone was unable to reliably distinguish tumour grade (SUV_max_ low-grade 2.03 and 2.18 for high-grade, p = 0.64). The biopsies were taken using a robotic arm with a biopsy needle as opposed to a craniotomy for tumour resection as in the Pafundi study [57]. The result is that the preplanned targets and resultant biopsies are likely to have been more accurate as they would have not been affected by brain shift from opening the dura and CSF drainage as would have been the case with the Pafundi study [57]. However, the single trajectory resulted in two biopsy targets (an area of hypometabolism and hypermetabolism) that were in close proximity with little margin for error in coregistration of the patient with the imaging.

The majority of ^18^F-FDOPA-PET studies have used static parameters for ^18^F-FDOPA uptake. Static PET provides a single snapshot of the tracer uptake whereas dynamic PET takes multiple snapshots at different timepoints. The advantage of dynamic PET when used alongside kinetic modelling allows creation of time-activity curves (TAC) which can provide additional information on the pharmacokinetics of ^18^F-FDOPA in brain tumours. The assumption is that high-grade gliomas will quickly take up ^18^F-FDOPA (the wash-in) when injected in comparison to low-grade gliomas which will have a slow wash-in period [64]. Previously Schiepers et al. have directly compared static and dynamic ^18^F-FDOPA-PET in brain tumours [42]. In this study 37 patients were included of which 33 were primary brain tumours. A significant difference was found between tracer volume distribution between newly diagnosed low-grade and high-grade tumours (p ≤ 0.01) and between newly diagnosed high-grade tumours and tumours with post-treatment changes. Nioche et al. similarly performed static and dynamic ^18^F-FDOPA-PET/CT for 33 patients published in 2013 and found a SUV_mean_ threshold of 2.5 in determining the grade with a sensitivity of 94% and specificity of 66% [65]. There was no significant improvement in these results when comparing static and dynamic imaging. Dynamic ^18^F-FDOPA-PET appears to be more useful in newly diagnosed gliomas and less useful for detection of recurrence or progression as evidence by Zaragori et al. [66]. This study identified 51 patients with suspected glioma recurrence or progression who underwent ^18^F-FDOPA-PET and found that no additional significant information was gained from performing dynamic imaging. Xiao et al. in a recent meta-analysis reported a pooled sensitivity of 0.71 and specificity of 0.86 for grading newly diagnosed gliomas with static FDOPA PET [67].

Despite the sensitivity and specificity of static ^18^F-FDOPA-PET, dynamic ^18^F-FDOPA-PET has the advantage of being able to detect a tumour’s molecular characteristics as demonstrated by Ginet et al. [68]. This retrospective study looked at 58 patients with newly diagnosed glioma who underwent either biopsy (n = 24) or surgical resection (n = 34) and preoperative static and dynamic ^18^F-FDOPA-PET. Patients were given carbidopa one hour prior to PET imaging. They found that only the dynamic parameters of time-to-peak (TTP) which represents the time from tracer injection to maximum SUV, area under the curve (AUC) and curve slopes were significant in predicting the IDH-mutation status (TTP p ≤ 0.001, AUC p = 0.789, slope p = 0.013). For prediction of the 1p/19q co-deletion status the TTP was the only dynamic parameter found to be statistically significant (p = 0.034). The static imaging did not significantly correlate with the molecular subtypes. This could be because static imaging is just a snapshot in time whereas dynamic imaging captures the rate of ^18^F-FDOPA tracer uptake and washout so more information is available. Isal et al. retrospectively looked at 20 patients with histologically confirmed newly diagnosed grade II and grade III gliomas that had serial static ^18^F-FDOPA PET [69]. The authors found that a SUV_max_ of greater than 1.8 is predictive of the presence of an IDH-mutation. Similarly, Cicone et al. performed static ^18^F-FDOPA-PET/CT in 33 patients following surgery but before initiation of chemoradiotherapy [70]. The SUV_max_, TBR and TSR were not statistically significant between tumours that were IDH-mutant or IDH-wildtype and that no difference was found between 1p/19q co-deleted and non-co-deleted patients. In addition, no statistically significant difference was seen between low- and high-grade gliomas (p ≥ 0.2).

The presence of MGMT methylation indicates silencing of the MGMT gene resulting in a reduction in the capability of tumour cells to repair damage from alkylating agents such as temozolomide and confers a better prognosis [71]. Cimini et al. performed static ^18^F-FDOPA-PET/CT in 72 patients post-surgery and found no difference between the presence of MGMT methylation versus unmethylation (p = 0.15) and no difference between IDH-mutant and IDH-wildtype gliomas (p = 0.79) [72]. One explanation for the results in both these studies may be that the ^18^F-FDOPA-PET scans were performed following surgical biopsy and it has already been shown that inflammation and macrophage response seen post-surgery can alter ^18^F-FDOPA uptake [73, 74]. The ability to detect IDH-mutation status on imaging is novel and could potentially play a role in management discussions with patient’s at diagnosis as IDH-wildtype tumours regardless of grade have a shorter median overall survival, < 2 years, when compared to IDH-mutant gliomas [75].

The GLIROPA clinical trial form Girard et al. included only newly diagnosed diffuse gliomas of which 32 biopsies were acquired from 14 patients [56]. Preoperative static and dynamic ^18^F-FDOPA-PET was performed followed by stereotactically guided biopsies (up to three per patient). The PET/CT was performed 10 min after tracer injection and no carbidopa premedication was administered. Each biopsy sample was graded independently but there was a much higher number of high-grade tumour samples (n = 23) in comparison to low-grade tumour samples (n = 9). The authors established that the static PET parameters were not significantly different between grades but the kinetic analysis from dynamic image acquisition was more accurate for glioma grading. However, the time between the ^18^F-FDOPA-PET/CT and stereotactic biopsy to confirm the histopathological grade was as long as 110 days during which time the tumour had the potential to transform to a higher grade. Janvier et al. performed a retrospective review published in 2015 on 31 patients of which 6 were recurrent gliomas and 25 newly diagnosed who had undergone static ^18^F-FDOPA-PET [76]. The study found that the SUV_mean_ and tumour-to-normal-tissue ratio (T/N) best correlated to the grade (p =  < 0.05) with a cut-off for SUV_mean_ of 1.33. Bund et al. found that in a subset of low-grade gliomas, in discriminating between dysembryoplastic neuroepithelial tumour and grade II oligodendroglioma SUV_max_ was significant (p ≤ 0.01) and also between low-and high-grade gliomas a SUV_max_ cut off of 2.16 was found [77]. A prospective study in 45 patients with suspected glioma who underwent preintervention static ^18^F-FDOPA-PET and found that a T/N SUV_max_ ratio greater 1.7 was able to differentiate high-grade glioma from other graded lesions [78].

In a pilot study Ponisio et al. used dynamic ^18^F-FDOPA-PET/MRI with stereotactically linked histopathology data in 10 patients of which 4 were recurrent tumours, obtaining a total of 23 biopsies. The authors identified that the results of the ^18^F-FDOPA-PET/MRI had a positive impact on patient management in 4 cases including performing additional biopsies and altering surgical strategies in terms of extent of resection. In addition, the authors found a strong correlation between tumour SUV parameters and the Ki-67 index reflecting cell proliferation. Despite the promising results and impact on management Ponisio et al. concluded that dynamic ^18^F-FDOPA-PET/MRI was independent of WHO grade and did not significantly differentiate between low- and high-grade gliomas. This pilot study and Todeschi et al. [63] differs from the other studies with stereotactically linked histopathological data [56, 57] in that the ^18^F-FDOPA-PET was combined with MRI rather than CT. There are implications in terms of cost, reduction in radiation exposure and accessibility in using PET/MRI but they are reported to perform equally [79].

There has been a lot of work on the ability of ^18^F-FDOPA-PET to differentiate between low- and high-grade gliomas since the 2016 recommendations were published which have been discussed but there are a number of limitations to consider. Firstly, many studies include both newly diagnosed and recurrent gliomas together. Macrophages have been reported to have high levels of amino acid transport and therefore ^18^F-FDOPA uptake. As a result, ^18^F-FDOPA uptake levels may be falsely positive following surgery and radiotherapy due to the presence of macrophages around the resection cavity and in irradiated tumours [73, 74]. For this reason, ideally newly diagnosed and recurrent gliomas should be reviewed separately to exclude treatment bias and if not possible, the time delay between surgery and ^18^F-FDOPA imaging should be considered and any uptake around a resection cavity should be interpreted with care. Secondly, in the majority of studies the number of participants is generally low and there are differing protocols from the biopsy and surgical technique to the co-registration of PET/CT with MRI and histological interpretation of tissue samples and therefore it is impossible to completely merge data from different studies. In addition, there are few studies [56, 57, 63, 80] that have integrated biopsy location with histopathological data and ^18^F-FDOPA uptake. This is important as ^18^F-FDOPA uptake can vary widely within a tumour. The ^18^F-FDOPA uptake heterogeneity may represent heterogeneity in terms of the histopathological features and therefore grade, which can alter management strategies and ultimately prognosis. Finally, the current published studies are all single centre and there is a requirement here for multicentre studies to be completed.

## Future work

Additional larger prospective studies are needed with biopsy validated dynamic ^18^F-FDOPA-PET/CT and PET/MRI in treatment naïve gliomas to confirm the promising correlation seen in the literature. A stage 2 clinical trial run by the Mayo Clinic (Rochester, United States) has now closed to enrolment having recruited 72 patients. With the aim of determining ^18^F-FDOPA-PET thresholds for distinguishing low- from high-grade glioma [81]. The FIG (^18^F-FDOPA-PET PET imaging in glioma) study is currently recruiting patients in Oxford University Hospitals NHS Foundation Trust (Oxford, UK) with the aim of opening to recruitment at two further sites later this year. The FIG study is a feasibility study investigating ^18^F-FDOPA-PET and oxygen-enhanced MR guided histopathology in patients with suspected low-grade gliomas in a multi-centre setting [82]. The study will include up to 168 biopsies in 21 participants recruited from multiple neurosurgical centres in the UK. This study will be one of the first and largest study to use dynamic FDOPA PET/CT in determining grade of gliomas in a multi-centre setting.

The recent publication of the 2021 WHO classification of central nervous system tumours has incorporated numerous molecular changes that support the integrated diagnosis for each tumour type^1^ (Table 1). These additional molecular changes open up an opportunity for evaluation of dynamic ^18^F-FDOPA-PET in detection of molecular markers similar to the previous studies into IDH-mutation status, 1p/19q co-deletion status and presence of MGMT methylation [68–72] (See Table 2).

**Table 1:** Summary of spatially linked ^18^F-FDOPA-PET studies

Authors	Year	Number of patients	Number of newly diagnosed gliomas	Number of recurrent gliomas	Type of imaging	Number of biopsies performed	Type of study	FDOPA thresholds
Girard et al	2021	14	14	0	Dynamic PET/CT	32	Prospective	None given
Pafundi et al	2013	10	8	2	Static PET/CT	23	Prospective	TBR > 2.0 equates to high-grade components of astrocytic tumours
Ponisio et al	2020	10	6	4	Dynamic PET/MRI	23	Prospective	None given
Todeschi et al	2019	20	16	4	Static PET/CT	20	Prospective	SUVmax > 1.75 gave a higher yield for high grade

**Table 2:** Summary of non-spatially linked ^18^F-FDOPA-PET studies and the key findings

Authors	Year	Total number of patients	Number of newly diagnosed gliomas	Number of recurrent gliomas	Type of imaging used	Type of study	Key Findings
Fueger et al	2010	59	22	37	Static ^18^F-FDOPA-PET/CT	Combination of prospective + retrospective	^18^F-FDOPA SUV_max_ cut-off of 2.72 to distinguish low- and high-grade newly diagnosed gliomas
							No significant difference in recurrent tumours
Schiepers et al	2007	37	33	4	Static and dynamic ^18^F-FDOPA-PET/CT	Prospective	Significant difference between tracer volume distribution of newly diagnosed low + high-grade tumours (p ≤ 0.01)
Nioche et al	2013	33	20	13	Static and dynamic ^18^F-FDOPA-PET/CT	Prospective	SUV_mean_ threshold of 2.5 in determining the grade (sensitivity 94%, specificity 66%)
							No significant improvement when comparing static + dynamic imaging
Zaragori et al	2020	51	0	51	Dynamic ^18^F-FDOPA-PET/CT	Retrospective	No additional information gained from dynamic imaging
Ginet et al	2020	58	58	0	Static and dynamic ^18^F-FDOPA-PET/CT	Retrospective	TTP and AUC significant in predicting IDH-mutation status (TTP p ≤ 0.001, AUC p = 0.789, slope p = 0.013)
							TTP significant in prediction of 1p/19q co-deletion status (p = 0.034)
Isal et al	2018	20	20	0	Static ^18^F-FDOPA-PET/CT	Retrospective	SUV_max_ > 1.8 predictive of the presence of an IDH-mutation
Cicone et al	2019	33	33	0	Static ^18^F-FDOPA-PET/CT	Prospective	No significant difference seen between low- + high-grade gliomas (p ≥ 0.2)
Cimini et al	2020	72	Not commented on	Not commented on	Static ^18^F-FDOPA-PET/CT	Retrospective	No difference between presence of MGMT methylation versus unmethylation (p = 0.15)
							No difference between IDH-mutant and IDH-wildtype gliomas (p = 0.79)
Janvier et al	2015	31	25	6	Static ^18^F-FDOPA-PET/CT	Retrospective	SUV_mean_ + T/N ratio correlated to grade (p = < 0.05)
Bund et al	2017	53	53	0	Static ^18^F-FDOPA-PET/CT	Prospective	SUV_max_ cut off 2.16 significant for discriminating low- + high-grade glioma
Patel et al	2018	45	45	0	Static ^18^F-FDOPA-PET/CT	Retrospective	T/N SUV_max_ ratio > 1.7 able to differentiate high-grade glioma from other graded lesions

## Conclusions

The literature demonstrates the capability of ^18^F-FDOPA-PET to differentiate low- from high-grade gliomas as well as the presence of an IDH-mutation, the 1p/19q co-deletion status and the MGMT methylation status. The FIG multicentre study will possibly answer questions on the introduction of preoperative ^18^F-FDOPA-PET into the routine imaging work up for glioma patients to guide surgical management. Detecting areas of high-grade transformation more effectively would allow for earlier clinical intervention, optimisation of surgical planning and resection strategies with the potential to lengthen progression free survival and therefore ultimately prognosis.
